# A scoping review of the implementation and cultural adaptation of school-based mental health promotion and prevention interventions in low-and middle-income countries

**DOI:** 10.1017/gmh.2024.48

**Published:** 2024-04-12

**Authors:** Patricia Harte, Margaret M. Barry

**Affiliations:** WHO Collaborating Centre for Health Promotion Research, University of Galway, Galway, Ireland

**Keywords:** mental health promotion, prevention, schools, interventions, implementation, low-and middle-income countries, scoping review

## Abstract

Effective school-based mental health promotion and prevention interventions in low-and middle-income countries (LMICs) can positively impact the mental health and well-being of large numbers of young people. This scoping review aimed to investigate the implementation of effective mental health promotion and prevention interventions in LMIC schools. A scoping review of the international literature was conducted and followed the Preferred Reporting Items for Systematic reviews and Meta-Analysis extension for Scoping Reviews guidelines. Medline, PsycInfo, Scopus, Embase, CINAHL and Cochrane were searched for peer-reviewed literature published from 2014 to 2022. PsycExtra, Google Scholar and the websites of key organisations were searched for relevant grey literature. Study selection focussed on mental health promotion interventions, including the development of social and emotional skills and mental health literacy, and prevention interventions, including anti-bullying and skill-based interventions for “at-risk” students. Twenty-seven studies evaluating 25 school-based interventions in 17 LMICs were included in the review. Fifteen interventions were developed in the implementing country and 10 were adapted from high-income countries (HICs) or other settings. Findings from the studies reviewed were generally positive, especially when interventions were implemented to a high quality. Universal life-skills interventions were found to increase social and emotional skills, decrease problem behaviours and positively impact students’ mental health and well-being. Mental health literacy interventions increased mental health knowledge and decreased stigma among students and school staff. Outcomes for externally facilitated anti-bullying interventions were less positive. All 19 effective studies reported on some aspects of programme implementation, and 15 monitored implementation fidelity. Eleven studies outlined the programme’s underpinning theoretical model. Only four studies reported on the cultural adaptation of programmes in detail. Including young people in the adaptation process was reported to facilitate natural cultural adaptation of programmes, while input from programme developers was considered key to ensuring that the core components of interventions were retained. The review findings indicate increasing evidence of effective mental health interventions in LMIC schools. To facilitate the sustainability, replication and scaling-up of these interventions, greater attention is needed to reporting on intervention core components, and the processes of implementation and cultural adaptation in the local setting.

## Graphical abstract

**Figure:**
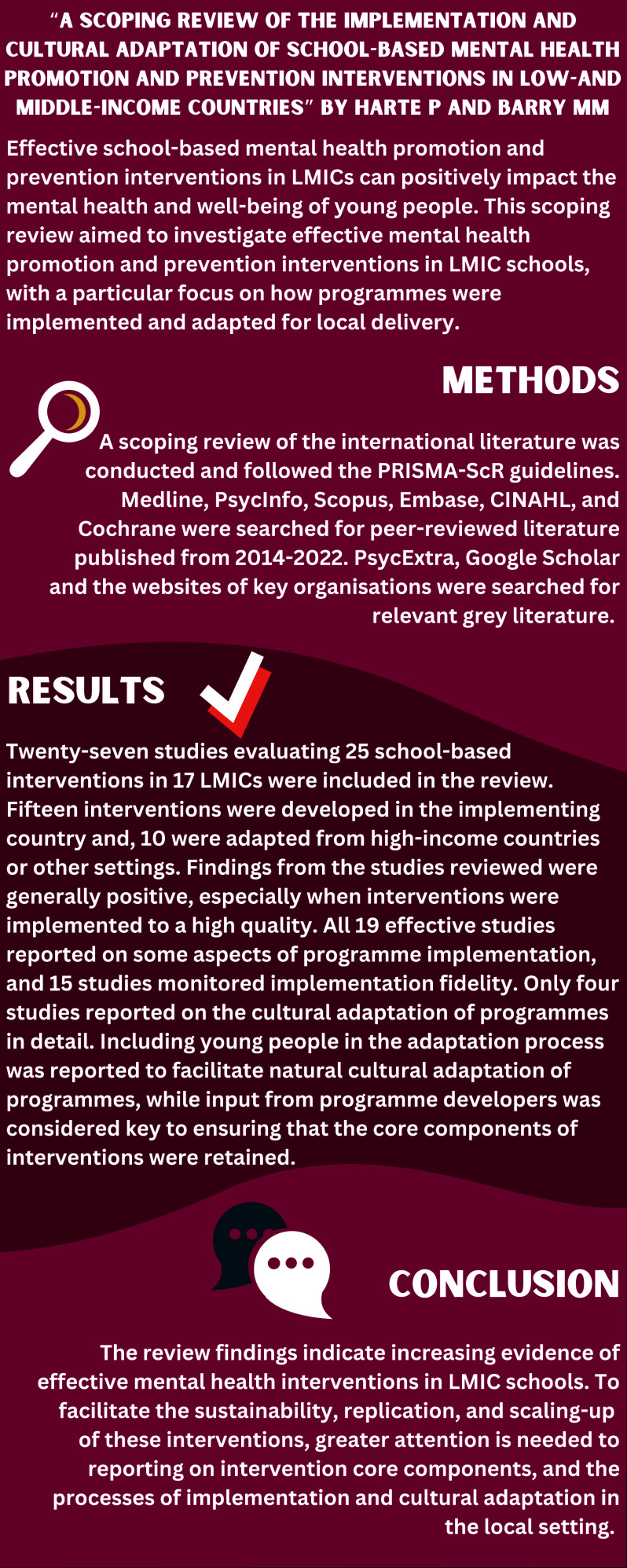

## Impact statement

Effective school-based mental health promotion and prevention interventions in low-and middle-income countries (LMICs) can positively impact the mental health and well-being of large numbers of young people. This scoping review sought to map the peer-reviewed and grey literature published from 2014 to 2022 on the process of implementing effective mental health promotion and prevention interventions in schools in LMICs. A total of 27 studies evaluating 25 school-based mental health interventions were identified. This review has a particular focus on how programmes were implemented and adapted for local delivery, thereby adding to the dearth of literature on implementation and the cultural adaptation of mental health interventions in LMICs. The increase in the number of studies reporting on implementation is encouraging, although reporting varied greatly between studies. Fifteen effective studies measured programme implementation, with quality of delivery being the most widely reported domain. The review findings endorse the importance of high-quality programme implementation to ensure positive outcomes. Findings also highlight the need to monitor and address barriers to implementation and to measure multiple domains of implementation, including the core dimensions; dosage, adherence, quality of delivery, participant responsiveness and programme differentiation. In addition, evidence-based interventions from HICs and other settings can be delivered effectively in LMIC schools when they are adapted to the local context. Cultural adaptation of interventions promotes participant responsiveness and the local acceptance of programmes. Reporting on the adaptation process facilitates the replication and scaling-up of interventions; however, only four of the reviewed studies reported on the cultural adaptation process in detail. The review findings highlight the need for a greater focus on supporting and reporting on the implementation process employed in under-resourced schools in LMICs, including the cultural adaptation of interventions using appropriate frameworks in a process involving young people and programme developers.

## Introduction

Good mental health is a basic human right and an essential component of overall health and well-being (WHO, 2022). Positive mental health is a necessary resource for optimal quality of life and is fundamental to the development of safe communities and sustainable global development (UN, 2015). Poor mental health adversely impacts individuals, families, communities and the economy (Renwick et al., 2022; WHO, 2022). It is estimated that 13% of adolescents globally aged 10–19 years are living with a diagnosed mental disorder, with many more reporting sub-clinical psychosocial stress (UNICEF, 2021), and suicide is the fourth leading cause of death among males and females aged 15–29 years (WHO, 2022). Evidence points to the increased prevalence of mental ill-health among young people since the COVID-19 pandemic (UNICEF, 2021; WHO, 2022), particularly among those living in low-and middle-income countries (LMICs) who face disproportionate levels of adversity (WHO, 2022). The explicit reference to mental health in the United Nations (UN) Sustainable Development Goal 3.4 (UN 2015) and recent global reports focussing on the importance of promoting young people’s mental health (UNICEF, 2021; WHO, 2022) endorse the need for effective mental health promotion and prevention interventions for young people, particularly in LMICs where 90% of the world’s young people live (World-Bank, 2017).

Mental health is shaped by a complex interaction of individual, family, community and structural level factors (WHO, 2012), and childhood and adolescence represent particularly vulnerable periods in mental health development (UNICEF, 2021). Poor mental health during these developmental periods adversely affects positive development, social behaviours, educational outcomes and the health of future generations (Renwick et al., 2022). Mental health promotion and prevention interventions effectively implemented during childhood and adolescence, particularly in school settings, can positively impact mental health and well-being, and increase social and emotional skills and the academic performance of young people (Durlak et al., 2011; Barry et al., 2013; Taylor et al., 2017; Aldridge and McChesney, 2018), including those who have been exposed to adverse experiences (Higgen et al., 2022).

Schools form a critical part of the socio-ecological system within which young people’s mental health develops and are therefore an important setting for mental health promotion (WHO, 2009, 2014; Barry et al., 2019). Universal skill-based interventions (Skeen et al., 2019; Singla et al., 2020; Chuecas et al., 2022) and social and emotional learning programmes (Durlak et al., 2011; Taylor et al., 2017) in schools promote positive behaviours and relationships, improve mental health and can improve academic performance; while mental health literacy interventions can encourage help-seeking behaviours and decrease stigma among students and school staff (Kelly et al., 2007; Yamaguchi et al., 2020). Additionally, since schools can be home to risk factors for poor mental health such as bullying and academic stress, school-based primary prevention programmes (universal, selective and indicated) can reduce the risk of mental ill-health (Harrison et al., 2022). Evidence points to the positive impact and cost-effectiveness of whole-school universal interventions that follow the World Health Organisation (WHO) Health Promoting Schools Framework (WHO, 2021b) over curriculum-only programmes (Peterson et al., 2016; Singla et al., 2020; WHO/UNICEF, 2021; Higgen et al., 2022); however, high-quality implementation is crucial to the effectiveness and sustainability of programmes (Durlak and DuPre, 2008; Durlak et al., 2011).

Implementation refers to the way in which programmes are delivered in real-life settings (Durlak, 2019) and is influenced by individual, community and macro-level factors (Domitrovich et al., 2008). Monitoring and reporting on the five inter-related domains of implementation, including adherence, dosage, quality of delivery, participant responsiveness and programme differentiation (Dane and Schneider, 1998), is crucial to the fair interpretation of outcomes and replication of programmes (Dowling and Barry, 2020; Singla et al., 2020). Exploring and addressing the barriers and facilitators to implementation is considered key to the sustainability and scaling-up of programmes outside of research conditions (Domitrovich et al., 2008). Previous studies have outlined various moderators of effective implementation (Domitrovich et al., 2008; Rojas-Andrade and Bahamondes, 2019). In low-resource schools in LMICs, barriers to effective implementation can compromise outcomes, including high pupil-teacher ratios (TISSA, 2014; McMullen and McMullen, 2018) and insufficient resources and funding that necessitate adaptations to programmes, such as shortening programme durations or delivery by volunteers (Strohmeier and Spiel, 2019). Research has also highlighted the importance of culturally adapting interventions to the local context and culture (Castro-Olivo, 2017; Bradshaw et al., 2021) using “a priori” frameworks (Peterson et al., 2017) to guide the process such as the ecological validity model (EVM) (Bernal et al., 1995) or Barrera and Castro’s (2006) four-step heuristic framework. Adaptations made to programme language, content and concepts, in a process involving key stakeholders can increase participant responsiveness and programme acceptance (Castro-Villarreal and Rodriguez, 2017; Catalano et al., 2019).

A research gap exists in the context of school-based mental health promotion and prevention interventions in LMICs as much of the available robust evidence is from HICs (Das et al., 2016; Peterson et al., 2016; Chuecas et al., 2022). Although recent literature suggests an increase in reporting on implementation in HICs (Dowling and Barry, 2020), few studies use quantifiable measures and/or report on all five domains of implementation (Hagermoser Sanetti and Fallon, 2011; Bruhn et al., 2015; Singla et al., 2020). Likewise, relatively few studies report specifically on how programmes from HICs and other settings can be culturally adapted for delivery in LMICs (Bradshaw et al., 2021). In addition, many existing evidence reviews focus primarily on studies employing randomised controlled trials (RCTs) and robust quasi-experimental study designs and search only electronic academic databases. While RCTs are considered the gold standard in assessing the internal validity of programmes, a mixed methods approach to evaluation can better determine their external validity (McQueen, 2001). A grey literature search and the inclusion of all study designs is important in the context of research from LMICs, to allow for a more complete mapping of the evidence, especially with regard to the implementation process and cultural adaptation of interventions (Gimba et al., 2020; Chuecas et al., 2022).

This review, therefore, aimed to investigate the implementation process involved in delivering effective mental health promotion and prevention (universal, selective and indicated) interventions in school settings for children and adolescents in LMICs. The inclusion of all study designs and a grey literature search allowed for better exploration of the study objectives.

Specific study objectives include:

- to investigate the primary outcomes of interventions on the mental health and well-being of participants and any secondary outcomes on physical health, knowledge, stigma and health behaviours;
- to investigate the number of effective studies that provided details of implementation;
- to examine the process of implementation of interventions;
- to identify any barriers or facilitators to the effective implementation of programmes;
- to detail any cultural adaptations made to programmes from their original models.

## Methods

A scoping review of the literature was conducted and followed the Preferred Reporting Items for Systematic reviews and Meta-Analysis extension for Scoping Reviews (PRISMA-ScR) (Tricco et al., 2018). The process was guided by the Arksey & O’Malley five-stage framework (Arksey and O’Malley, 2005).

### Eligibility criteria

Study selection criteria were developed in line with the population concept context (PCC) framework (Munn et al., 2018), which informed the research question. Academic and grey literature in electronic form published from 2014 to 2022 was deemed eligible for inclusion if: (i) participants were boys/girls attending primary/secondary schools, (ii) interventions were school-based and aimed to promote positive mental health/prevent mental disorders of participants and (iii) interventions were implemented in LMICs as classified by the World Bank Criteria (World Bank, n.d.) at the time of the study (https://datahelpdesk.worldbank.org/).

All primary studies, including RCT, quasi-experimental, cohort and qualitative study designs, were eligible if they met the inclusion criteria. Primary outcomes of interest concerned the mental health and well-being of participants and any secondary outcomes on physical health, knowledge, stigma and health behaviours were noted. Studies evaluating interventions delivered in humanitarian settings and targeting young people with specific disabilities, for example, children with stuttering deficit, were excluded. Due to time constraints, they were considered special cases and beyond the scope of this review.

### Search strategy

The electronic academic databases Medline, PsycInfo, Scopus, Embase, CINAHL and Cochrane were searched in May 2022 for relevant peer-reviewed articles. PyscExtra, Google Scholar and key websites (WHO and UNs International Children’s Emergency Fund [UNICEF]) were also searched for relevant grey literature. Search limiters included English language texts only, published between 2014 and 2022. This specific time frame was chosen to map the evidence since the publication of the WHO Comprehensive Mental Health Action Plan in 2013 (WHO, 2021a). In addition, the reference lists of key studies were hand-searched to ensure no relevant studies were missed.

Two concept searches were conducted in each database. Individual search terms were combined with the Boolean Operator “OR” and concepts combined with “AND” (see Supplementary Tables S1 and S2 for the search terms and concepts used across all databases and the specific search strategy used in CINAHL database, respectively).

Search 1: (population terms) AND (setting terms) AND (positive mental health terms) AND (programme terms) AND (context terms).

Search 2: (population terms) AND (setting terms) AND (negative mental health terms) AND (programme terms) AND (context terms).

### Study selection

**Figure 1.**
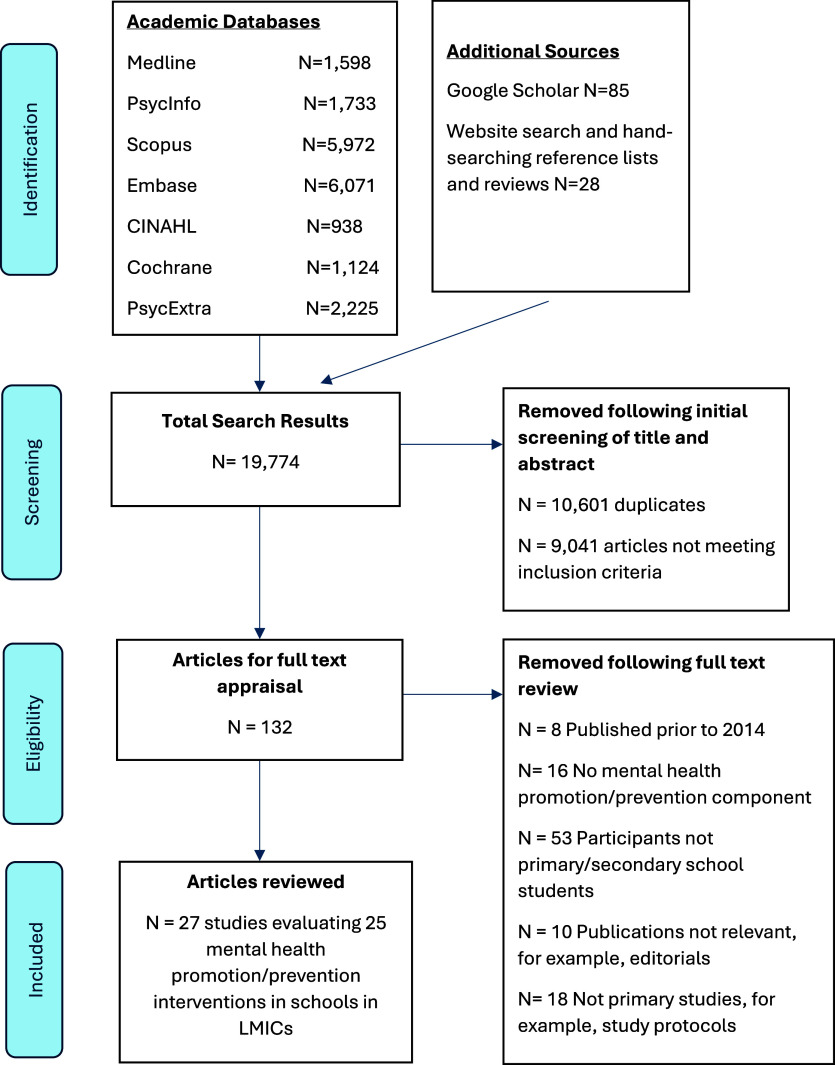
PRISMA diagram.

The search process yielded 19,746 studies. After deduplication, 9,537 studies remained and were exported to Rayyan software (Ouzzani et al., 2016), where further deduplication left 9,145 articles. Following screening of those titles and abstracts using the inclusion/exclusion criteria outlined, 104 articles remained for full-text review. A further 28 eligible studies were identified from website searches and by hand-searching key studies, leaving a total of 132 studies for full-text review. The final selection of studies for inclusion was based on a review by two researchers (PH and MMB). Twenty-seven studies that met the eligibility criteria were selected for data extraction (Figure 1 contains a PRISMA flow chart summarising the search and screening process).

### Data extraction

Studies were grouped according to the type and focus of the intervention. Data were then extracted, sorted and charted in tabular form in Microsoft Excel. The data extracted reflected the study aim and objectives. A narrative synthesis of results was then undertaken, which provided a commentary on the main findings.

Although critical appraisal of studies is not recommended in scoping reviews (Peters et al., 2020; Pollock et al., 2023), quality appraisal was carried out to give more depth to the discussion and was guided by Joanna Briggs Institute checklists (JBI, 2020), available at https://jbi.global/critical-appraisal-tools. Checklists were selected as appropriate for the type of study design under review and studies were rated strong, moderate, or weak.

## Results

Based on the review process, 27 studies evaluating 25 school-based mental health promotion and primary prevention interventions in 17 LMICs were identified (see Tables 1–3). Study designs included RCT (N = 6), cRCT (N = 4), quasi-experimental (N = 7), pre-post design (N = 5), cohort (N = 1), qualitative (N = 1), pseudo-random (N = 1), two-group comparison (N = 1) and one was a 2-year follow-up cross-sectional study. Five studies were feasibility or pilot studies. Study quality varied with 10 studies receiving a strong quality rating, 11 receiving a moderate rating, while four studies received a weak rating. Sample sizes varied widely between studies from N = 29 to N = 10,202.

Of the 25 interventions reviewed, 13 were considered universal mental health promotion programmes, five contained both promotion and prevention elements, six were considered primary prevention programmes and one intervention was a whole-school, multi-component health promotion intervention. The focus of the mental health promotion interventions varied and included the development of social and emotional skills, positive psychology, mindfulness and resilience, while two interventions focussed specifically on mental health literacy (see Table 1 for details). Interventions incorporating both promotion and prevention elements were skill-based or focussed on stress-reduction (see Table 2 for details). Prevention programmes focussed on anti-bullying, life-skills for “at-risk” students (Guzmán et al., 2015), cognitive behavioural therapy for adolescents with subclinical depression (Singhal et al., 2018) and yoga to prevent depression, anxiety and aggression (Velasquez et al., 2015) (see Table 3 for details).

**Table 1.** School-based mental health promotion interventions in LMICs

Study name, focus of intervention, location, author and year	Target group	Type of intervention, modules, duration and facilitator details	Study design, date (if provided), sample details	Outcomes	Implementation details	Cultural adaptation details (where provided)	Key findings
Reaching Educators, Children and Parents–Vietnam (RECAP–VN) Skill–based Vietnam (Dang et al., 2017)	Second grade students in three primary schools	Universal social skills programme with classroom behaviour management system Original programme: RECAP United States (US), for school and home (Han et al., 2005) Modules: social skills, reattribution, communication skills, self–control, affect recognition and expression and relaxation Implemented twice per week over 16 weeks throughout one academic year Facilitators: teachers	RCT (pilot study), 2013–2014 N = 443 Controls: Received no intervention	Positive effects for: Assertive behaviour and self–control (low–risk participants only, measured at midpoint) Emotional internalising mental health problems and behavioural externalising problems (measured post–intervention) No effects for: Cooperation or empathy	Teachers took part in a 1–day, on–site workshop on mental health literacy and classroom management pre–implementation. RECAP–VN consultants co–facilitated both weekly sessions for the first 2 months and one session per week thereafter Implementation quality monitored weekly by RECAP–VN consultants Monthly meetings held between teachers and RECAP–VN consultants Delivery as a universal class–based intervention without parental involvement to non–exam classes facilitated implementation	Adaptations included translation of RECAP (US) to Vietnamese by psychologists, the original author and Vietnamese teachers	Social skills outcomes significant for low–risk participants only Mental health outcomes significant for both low and high–risk groups Effects sizes larger for social skills outcomes than mental health outcomes
Programa Compaso (PC) Skill–based Brazil (McCoy et al., 2021)	Third and fifth grade students (average age = 9.85 years) in 90 primary schools	School–wide SEL intervention Original programme: Second Step SEL (US) (Frey et al., 2000) Modules: emotion identification, executive function, empathy, social problem solving Implemented once per week (50 min) through 22 sessions over one academic year Facilitators: teachers	RCT, 2017 N = 3,018 Controls: Received standard curriculum	No significant effects for: Behaviour problems (teacher– reported) Emotion knowledge Executive function Small positive effect for: Emotional expression Inhibitory control (low–homicide communities only)	Materials required included puppets, videos and posters “Train the trainer” model used. NGO, Instituto Vila Educacao (IVE), provided an 8–hour training to a principal and teacher from each school who then trained their peers. One–hour monthly refresher courses delivered by IVE staff. Programme manual provided Implementation was monitored through voluntary surveys completed by teachers post–intervention (52.22% response rate). Low implementation fidelity reported Implementation barriers included high levels of community unrest resulting in a high turnover of teachers	Adaptations included the translation of Second Step SEL Program (US) to Portuguese and the addition of extra components, such as a student workbook and references to football Process facilitated by IVE and included an initial pilot in 17 schools	Null effects attributed to implementation challenges and participants considered too high risk
Living Well Uganda Skill–based (McMullen and McMullen, 2018; McMullen and Eaton, 2021)	Senior Three and Five students (13–18 years) in four secondary schools in deprived areas	Life skills intervention Four themes included: living well with ourselves and others, living well with worry and stress, living well with life’s issues and living well in the future Implemented once per week (45–60 min) through 24 sessions over one academic year Facilitators: teachers	Cluster Randomised Trial (cRCT) N = 620 Wait–list controls and Qualitative Study	Significant increase in: General self–efficacy Overall connectedness (to self in present and future) Significant reduction in: Internalising problems (depression/anxiety symptoms) No significant effects for: Prosocial attitudes Connectedness to school, peers, or religion *only 27% (*n* = 170) of participants completed pre– and post–intervention questionnaires	Skills taught using discussion and problem solving, reflection and homework assignments. Materials required included a blackboard and one notebook per student Three days training was provided for teachers pre–intervention. Training expenses covered by NGO, Fields of Life (FOL). “Train–the–trainer” model used to train teachers from waitlist schools. Programme manual provided Implementation fidelity was monitored through monthly teacher reports that recorded the number of lessons completed, adherence and classroom management Implementation barriers included large class sizes	Lessons and questionnaires were delivered in English but translated to local dialect at the teachers’ discretion	Medium effect sizes, however, lack of randomisation and large number of participants lost to follow–up reported Low cost and “train–the–trainer” model offer potential for scaling–up in LMICs
Life skills, empowerment intervention Skill–based India (Sarkar et al., 2017)	Grades 6–9 students (11–17 years) in two rural schools in a tribal area	Life skills, education–based empowerment intervention Original programme: NIMHANS model of health promotion interventions (Bharath and Kishore, 2010) Modules: basic life skills and general health modules including goal setting, discipline, nutrition, hygiene, relationships, self–awareness, sexuality, social responsibility Implemented twice per week (45–120 min) on two successive days over 24 months Facilitators: researcher	Quasi–experimental study, using Solomon four–group design, 2013–2015 N = 742 Controls: Received no intervention	Significant improvement in: Resilience Internal health locus of control Self–determination Pathological behaviour *Measured 3 months post–intervention	Programme developed in consultation with local education and medical experts, following identification of life skills needs by teachers Activities included icebreakers to start lessons and games Implementation quality was monitored through session observations. 100% attendance by students. High implementation fidelity reported.	Programme translated to Bengali References to local dietary practices included	Positive effects, with improvements in resilience more significant for tribal adolescents Delivery by class teachers should be considered if scaling–up
The African Guide (AG) Mental health literacy Tanzania (Kutcher et al., 2017)	Secondary school students in 29 schools	Mental health literacy programme Original programme: “The Guide” (Canada) (Kutcher and Wei, 2014) Implemented over 1 year Facilitators: teachers	Pre–post impact evaluation N = 4,657 students N = 32 teachers	Improvement in (100% of students, teacher–report): Knowledge about mental health and illness Attitudes towards people with mental illness Behaviour towards people with mental illness Improvement in (teachers): Knowledge about mental health and illness (83.3% of teachers) Behaviour towards people with mental illness (87% of teachers) *teacher attitude data not available due to transcription error	“Train the trainer” model used where trained teachers facilitated training for peers. Training provided to teachers pre–intervention and 6 months later Implementation fidelity assessed by teacher self–report surveys at 6, 10 and 12 months. Surveys assessed the number of classes in which the AG was taught, number of students exposed to AG, number of teachers trained in AG by original teachers, number of students approaching teachers about their mental health and number referred for further healthcare (M = 4.50 referrals per teacher)	Adapted by Malawian and Tanzanian mental health experts	Positive results though outcomes were teacher reported and data around teacher attitudes missing Low–cost and “train–the–trainer” model offer potential for scaling–up in LMICs
“The Guide” Mental health literacy Study 1: Vietnam Study Two: Cambodia (Nguyen et al., 2020)	Study 1: Students in 20 secondary schools Study Two (pilot): Grades 7 and 11 students in one secondary school	Study 1: Mental health literacy programme Original programme: “The Guide” (USA Edition: Washington State, 2nd edition of (Kutcher and Wei, 2017) Modules included: stigma, understanding mental health, information on mental illness, experiences of mental illness, finding support, positive mental health Implemented twice per week (45 min) over 5 weeks Facilitators: teachers Study Two: As per Study 1, except implemented once per week (60–90 min) through six sessions over 8 weeks Facilitators: teachers	Study 1: RCT N = 2,539 students N = 80 teachers Controls: Received standard life skills curriculum Study Two: Quasi–experimental N = 275 students N = 67 teachers and non–teaching school staff Controls: Received standard curriculum	Study 1: Teachers: Positive effects (moderate to large effects sizes) for: Recognition Self–efficacy Willingness to interact Lower levels of stigma Beliefs Towards Mental Illness (2/3 subscales) Students: Positive effects (small effect sizes) for: Mental health knowledge Lower levels of stigma Study Two: Staff (excluding facilitating teachers): Positive effects for: Willingness to interact Beliefs Towards Mental Illness (all subscales) Students: Positive effects for: Mental health knowledge Lower levels of stigma	Study 1: Training provided over 3 days pre–intervention (2 days on mental disorders and stigma and 1 day on lesson planning). Teachers paid for training and delivering the course. Study Two: All staff received the 2–day course and only facilitating teachers received the third day of training Implementation quality was monitored and rated through weekly lesson observations Study 1: High fidelity in all except Correct Process (deemed satisfactory) Study Two: Satisfactory scores in all except Student Participation (below satisfactory) Implementation facilitators included delivery during life skills class Implementation barriers included lack of ongoing support for teachers	Study 1: Two–stage cultural adaptation process: Stage One: Core concepts of English version examined by the research team for appropriacy and translated to Vietnamese Stage Two: Vietnamese version assessed by psychiatrists, teachers and psychologists and adapted. Adaptations made to mental illness terms and references to Vietnamese celebrities added Study Two: Translation to Khmer	Positive effects for decreased stigma among staff and students and improved mental health knowledge among students Need for more validated measurement scales Low cost and suitability for “train–the–trainer” model offer potential for scaling–up in LMICs
Mindfulness intervention Mindfulness India (Modi et al., 2018)	Grades 5–8 students (10–14 years) in a semi–urban, international school	Mindfulness intervention Modules included: introduction to mindfulness, meditation, breathwork, body scans Implemented once per week (40 min) over 10 weeks Facilitator: school psychologist (groups of nine–11 students)	Pre–post study N = 100 Controls: Received no intervention	Significant improvement in: Self–regulation Self–esteem Psychological wellbeing Mindfulness	Modules taught through mindful sitting/eating/walking/listening, body scans, group discussion and homework assignments		Positive effects for four indicators including psychological wellbeing, however small sample size
M–SEL Mindfulness/SEL Brazil (Waldemar et al., 2016)	Fifth grade students (10–14 years) in three public elementary schools	Combined Mindfulness and SEL Programme Underpinning Theoretical Frameworks: Mindfulness, CASEL competencies (CASEL, 2013) and The Council Method of the Ojai Foundation (Zimmerman and Coyle, 1996) Modules included: managing emotions, caring for and respecting others, positive relationships, behaving responsibly and decision making Implemented through eight–12 sessions (60 min) over 5 months Facilitators: volunteer, trainee psychologists	Quasi–experimental (feasibility study), pre–post design N = 132 Waitlist controls	Positive effects for: Emotional, conduct, relationship and prosocial behaviours General and total quality of life No effects for: Personal, relational, or environmental quality of life Attention deficit and hyperactivity	Modules taught through mindful breathing and activities, the “fishbowl technique” for developing positive relationships and the “collaborative chair game” Implementation quality monitored through weekly session observations		Positive effects for four mental health symptoms and general and total quality of life
Girls First Resilience Curriculum (RC) Positive psychology/ Resilience–based India (Leventhal et al., 2015)	Seventh and eighth grade girls (9–18 years) in 57 rural schools in marginalised areas	Resilience curriculum, delivered independently or with Girls First Health Curriculum (HC) Underpinning Theoretical Models: Positive psychology, emotional intelligence, restorative practices Modules included: listening skills, character strengths, life stories/goals and planning, emotional awareness, managing emotions and communication, conflict resolution, forgiveness/problem–solving, peace project and a review session Implemented once per week (60 min) over 23 weeks Facilitators: trained local women (groups of 12–15 girls)	RCT N = 2,308 Intervention: Received RC or RC + HC Controls: Received standard curriculum	Positive effects for: Emotional resilience Self–efficacy Social–emotional assets Psychological well–being Social well–being No significant effects for: Depression Anxiety	Modules taught through discussions, reflective listening, visualisation exercises, games and group projects Five days training pre–intervention and a three–day top–up midway through for RC facilitators. HC facilitators received 3 days training pre–intervention and a three–day top–up midway through. Programme manuals provided Implementation quality monitored bi–monthly through session observations. 85.4% adherence to session structure, 87.2% covered content adequately or better, 81.3% presented information clearly, 95.8% managed discipline, 91.7% maintained participants interest, 70.8% used participatory teaching methods. Participant attendance recorded	Programme delivered in Hindi Participants chose topics relevant to them for discussion, allowing for natural cultural adaptation	Medium to large effects for psychosocial assets. Small to medium effects for two aspects of psychosocial well–being Need to consider culturally relevant measures of psychosocial well–being
Global Resilience Oral Workshops (GROW) Positive psychology/ Resilience–based Zambia (Seale et al., 2021)	Grade 5 and 7 children (10–13 years) in 21 schools	Spiritually based, character strengths, resilience curriculum Original programme: GROW (US) (Seale, 2014) Underpinning Theoretical Models: Positive psychology and spirituality Implemented once per week (90 min) over 24 weeks, after school hours Facilitators: trained community leaders (groups of 20–24 students), co–led by a teacher	cRCT (pilot study), mixed methods evaluation N = 643 Intervention (Phase One): (July 2018–February 2019) Controls (Phase Two): Delayed–start (February–November 2019)	Positive effects for (Phase One group only): Psychological resilience at T2 &T3 Hope scores at T2 Meaning between T2 & T3 Gratitude between T2 & T3 Gratitude to God between T2 & T3 Negative effects for (Phase One group only): Meaning in life at T2 Daily spiritual experiences at T2 *T2 data collected in February 2019 *T3 data collected in November 2019	24 character strengths, for example honesty, forgiveness, perseverance, taught through bible storytelling, drama, prayer, games, dance and meditation. Materials needed included flip charts, markers, paper and crayons, notebooks, snacks for children, t–shirts for GROW graduates, badges for GROW Wings participants and mobile phones for leaders Two days interactive training provided pre–intervention, weekly training sessions and a booster session provided between phases. Programme manualised Implementation monitored through lesson observation, uploading of audio recordings of sessions on a WhatsApp group and submission of weekly checklists. Participant attendance varied widely between schools (mean of 79% attending at least 20 sessions). Decreased leader attendance at training in Phase Two. High fidelity reported	Adaptation involved initial consultation with all stakeholders, 3–week pilot and final analysis Adaptations included the addition of a programme motto, local customs, an object lesson and a GROW Wings components so Phase One participants could remain involved after completion. Bible verses and questionnaires were shortened	Positive effects for psychological resilience for Phase One group only
Positive Psychology Intervention Positive psychology Egypt (El–Abbassy et al., 2020)	Second– and Third–Year preparatory school students	Positive psychology intervention to promote life satisfaction, happiness and mental health Modules: gratitude, kindness, identifying character strengths, future self and goal mapping Implemented once per week (60 min) over 6 weeks, plus one introductory session Facilitator: researcher (groups of 20 students)	Quasi–experimental, One–group design, 2019 N = 80	Significant reduction in: Psychological distress symptoms Significant increase in: Self–efficacy Optimism Self–esteem Life satisfaction Happiness	Modules taught through storytelling, art, visualisation and homework assignments. Every module contained a loving–kindness meditation and savouring exercise		Positive intervention effects, though small sample size
Positive Education Intervention Positive education China (Zhao et al., 2019)	Eighth grade students in one public middle school	Positive education programme Underpinning Theoretical Model: Broaden–and–Build theory (Fredrickson, 2001) Modules: knowing and expressing emotions, gratitude, serenity, anxiety and anger Implemented once per week (45 min) over 10 weeks Facilitator: teacher	Pseudo–random experiment design N = 173 Controls: Received standard curriculum	Depression symptoms increased for both groups though significantly less so for the intervention group	Modules taught through meditation, breathing exercises, charades, “three good things” method, mindful eating, learning and practicing anger management skills Teacher trained in positive psychology and regularly met with researchers. Curriculum provided		Depression scores increased for both groups, however, the intervention showed small but positive protective effects against depressive symptoms
DepisNet–Thai Web–based mental health promotion Thailand (Anttila et al., 2019)	High school students of low socio–economic backgrounds (15–19 years)	Web–based programme to support mental well–being Underpinning Theoretical Model: Adolescent coping (Garcia, 2010) Modules: psychological stress, physical well–being, relationships with family/friends/society Implemented over five sessions (50 min) plus an additional two–week orientation Facilitators: teachers (groups of 11–14 students)	Parallel quasi–experimental cluster design (feasibility study), 2013 N = 180 adolescents Intervention group: Received intervention in groups Active controls: Used DepisNet–Thai independently Passive controls: Received no intervention	No significant effects for: Depression Stress	DepisNet–Thai was developed based on needs assessment with students Teachers received training pre–intervention. Programme manual provided Implementation barriers included technical difficulties setting up online accounts		No significant intervention effects for depression or stress Partial completion of the programme by participants was considered to have adversely affected outcomes
SEHER Whole–school, health promotion intervention Bihar, India (Shinde et al., 2018, 2020)	Grade 9 and 10 students (13–15 years) of 74 government–run secondary schools	Whole school, multi–component health promotion intervention (SEHER) delivered in addition to existing Grade 9 life–skills programme (AEP) Underpinning Theoretical Model: Health Promotion Whole–school, group and individual intervention activities delivered. Modules: mental health, bullying, hygiene, substance use, gender and violence, reproductive and sexual health, study skills Two–year programme Facilitators: lay counsellors (SM) or teachers (TSM)	3–arm cRCT N = 10,202 Intervention groups: Received SEHER (SM) or SEHER (TSM) Controls: Received AEP only & 2–year follow–up, cross–sectional study N = 15,232	Positive effects (SEHER (SM) only) for: School climate scores Attitudes towards gender equity Knowledge of sexual health Reduction in (SEHER (SM) only): Severity of depressive symptoms Frequency of bullying (self–report), violence victimisation and perpetration *Effects sustained and larger at 2–year follow–up for SEHER (SM) only	One week training pre–intervention and monthly in–service training provided to facilitators Implementation quality monitored through monthly logs completed by facilitators and 3–monthly observations by supervisors. SEHER (SM) more effectively implemented. Implementation barriers to SEHER (TSM) included time constraints		Positive and sustained effects for SEHER (SM) only. Intervention effects stronger for females No positive effects SEHER (TSM) Estimated cost of intervention: SEHER (SM) $15.0/student, SEHER (TSM) $7.4/student

**Table 2.** School-based mental health interventions containing both promotion and prevention elements in LMICs

Study name, focus of intervention, location, author and year	Target group	Type of intervention, modules, duration and facilitator details	Study design, date (if provided), sample details	Outcomes	Implementation details	Cultural adaptation details (where provided)	Key findings
Life skills intervention for suicide prevention Skill–based Mexico (Arenas–Monreal et al., 2022)	First year middle school students (12 years)	Life skills intervention for suicide prevention with family, school staff and community involvement Modules for students: self–awareness, empathy, communication and coping with emotions Implemented once per week (50–60 min) over 12 weeks Families received six sessions, staff received three sessions, community gatekeepers received 11 sessions Facilitators: researchers	Quasi–experimental, 2017–2018 N = 29 students N = 17 parents N = 18 school staff N = 23 gatekeepers No controls	Students: Significant increase for: Self–awareness Coping with emotions (females only) Parents/Guardians: Significant change in: Attitude towards adolescent suicide behaviour Devoting attention to the student Preparedness Academic Staff: Significant change in: Attitude concerning preparedness Community members: Significant improvement in: Prosocial behaviour Understanding of depression	Student sessions were class–based. Family and staff sessions took place in the school library. Community gatekeeper sessions took place in the Town Hall in conjunction with Patrulla Juvenil (Youth Patrol) 100% student attendance reported		Positive outcomes for participants, families, school staff and community gatekeepers
Life skills intervention Skill–based Cambodia (Jegannathan et al., 2014)	Secondary school students in two semi–urban schools	Universal life skills intervention for suicide prevention Original programme: “The Activity for the Teachers on Health Promotion Using Life Skills Approach” (Bharath and Kishore, 2010) Modules: concentration/memory, problem–solving skills, peer pressure, coping with stress, self–esteem, self–awareness and understanding depression and suicide Implemented once/twice per week (90–100 min) over 20–24 weeks Facilitators: teachers, psychologists, psychiatric nurse and school nurse	Pre–post design N = 299 students Controls: Received three general health sessions	Improvement in (girls): Interpersonal communication Physical Fitness Total Life Skills dimension Improvement in (boys): Interpersonal communication Improvement in (high–risk boys only): Interpersonal communication Identity development Total Life Skills Dimension Decrease in (high–risk boys only): Depression Attention problems Rule breaking behaviour Aggressive behaviour Externalising syndrome	Modules taught through discussion and homework assignments. Original programme shortened to focus on modules related to suicide risk factors		More significant improvement in life–skills dimensions for girls overall and males within the high–risk category No significant effects on mental health outcomes apart from high–risk males
The Positive Child and Youth Development Programme (Red Ball Child Play) Skill–based Pakistan (Karmaliani et al., 2020)	Grade 6 students (12–14 years) in 40 single sex government schools	Play–based, life skills intervention to decrease peer violence and depression and promote gender equality Underpinning Theoretical Model: Social, cognitive, child development and experiential learning Play–based learning activities followed by three–step discussion (Reflect–Connect–Apply framework) Implemented twice per week (35 min) through 120 sessions over 2 years Facilitators: male and female adult coaches employed by NGO, Right to Play	cRCT, 2015–2018 N = 1752 Waitlist controls	Reduction in (males and females): Peer–victimisation Peer–perpetration Self–reported depression Improvement in (males and females): Gender attitudes Decrease in (more significant for girls): Corporal punishment Physical punishment at home No significant effects for: School performance (self–assessed) School attendance	Programme developed by educationists, athletes, teacher–trainers and psychologists. Previously implemented in other LMICs Teacher training around positive youth development and child protection provided by coaches. Parental involvement through sports tournaments and 3–monthly awareness sessions around child rights and gender equality Implementation quality monitored by NGO staff and research partner, Aga Khan University, through monthly lesson observations and recording the number of sessions delivered	Original programme translated into Urdu and Sindhi	Positive effects for peer–victimisation, peer–perpetration and depression scores Improvements in gender attitudes and reductions in corporal punishment and violence at home (more significant for girls) Large sample size, 2–year study
ERSAE–Stress–Prosocial (ESPS) intervention Stress–reduction Tanzania (Berger et al., 2018)	Grade 4–6 primary school students	Universal stress–reduction and prosocial intervention Original Programme: ESPS implemented in humanitarian contexts (Gelkopf and Berger, 2009) Underpinning Theoretical Model: CBT and SEL Modules: stress–reduction (understanding stress, calming the body, coping with emotions, building social support, optimism) and prosocial (social skills, empathy, critical thinking) Implemented twice per week (45 min) over 16 weeks Facilitators: teachers	RCT, 2013–2015 N = 183 students Controls: Received standard curriculum	Significant increase in: Prosocial behaviour Children functioning Academic performance Significant decrease in: Social difficulties Hyperactivity Somatisation Anxiety Frequency of disciplinary behaviours *sustained at 8–month follow–up	Topics taught through experimental work, body work, contemplation, skill–learning and home–work assignments Four days training for teachers, facilitated by author, pre–intervention. Programme manual provided Implementation quality monitored and rated through bi–monthly classroom observations. High fidelity reported	Adaptation process involved local stakeholders. Programme piloted to check for cultural appropriacy Adaptations included translation to Kiswahili, allocation of extra time to learn skills, emphasis on body work, the inclusion of traditional folk stories, concepts and Kiswahili proverbs and traditional healing rituals by a local healer	Positive, though small effect sizes, for overall child functioning, prosocial behaviour and school adjustment Academic outcomes more significant for children living in an orphanage “Train–the–Trainer” model and low cost offer potential for scaling–up in LMICs
Three–stage mental health promotion programme Stress management Tabriz, Iran (Heizomi et al., 2020)	Grade 9 students in two single sex (female) schools	Multi–component, mental health promotion programme with stress management curriculum, changes to the school environment and “Joyful Programme” Underpinning Theoretical Model: McNamara Model (stress management curriculum) Modules: defining stress, exploring the impact of stress, coping strategies, strengthening self–esteem and relaxation techniques Implemented once per week (45–60 min) over 6 weeks Facilitator: clinical psychologist	Quasi–experimental N = 284 Controls: Received routine mental health measures	Positive effects for: Life satisfaction Happiness Perceived stress *both intervention and control groups, but more significant for intervention group No significant effects for: Psychological well–being Self–efficacy Hopefulness	Stress management curriculum taught through weekly lectures and practice. School environment changes included playing music during breaks. Joyful Programme included “tug–of–war” competitions to promote physical activity and cooking competitions to encourage healthy eating. Programme developed following needs assessment. Student participation rate was 98.2%		Positive effects found for this whole–school approach to mental health promotion

**Table 3.** School-based primary mental ill-health prevention interventions in LMICs

Study name, focus of intervention, location, author and year	Target group	Type of intervention, modules, duration and facilitator details	Study design, date (if provided), sample details	Outcomes	Implementation details	Cultural adaptation details (where provided)	Key findings
Viennese Social Competence (ViSC) Anti–bullying Kosovo (Arënliu et al., 2020)	Seventh and Eighth Grade students in nine schools	Short (6 weeks) and ultra–short (4 weeks) ViSC, anti–bullying programme Original programme: ViSC whole–school programme, Austria (Strohmeier et al., 2012) Modules: social skills (units one to four) and project work (units five and six) Implemented once per week over four or 6 weeks Facilitators: volunteer undergraduate students, supported by school psychologists	Quasi–experimental, 2017 N = 1,249 students N = 282 short version N = 354 ultra–short version N = 613 Controls: Received no intervention	Significant effects for: Physical victimisation (ultra–short programme) No significant effects for: Perpetration Cyber/bullying/relational/ verbal victimisation	Materials needed included flipcharts, markers, fingerpaints. Training provided to facilitators pre–implementation by Austrian programme developers. Programme manual provided Implementation quality monitored through facilitator reports. Implementation facilitators included adaptation to a class–level intervention, shortening the programme and previous implementation in LMICs. Implementation barriers included large class sizes and limited physical space		Positive effects for one victimisation indicator only for the ultra–short programme. Shortening the programme may have compromised its effectiveness Teacher–led, whole–school approaches to anti–bullying interventions implemented over longer periods should be considered
Skill–based, cognitive behavioural intervention *Selective, skill–based Brazil (Da Silva et al., 2016)	Sixth Grade victims of bullying in six schools	Cognitive behavioural intervention to reduce bulling victimisation among bullying victims Underpinning Theoretical Model: CBT Modules: civility, making friends, empathy, self–control, expressing emotion, assertiveness, inter–personal problem–solving Implemented once per week (50 min) over 8 weeks Facilitators: clinical psychologists (groups of eight to 10 female and male students)	Pre–post study design, 2015 N = 78 bullying victims N = 110 bystanders Comparison group (same school): No intervention	Significant decrease in (intervention and comparison groups): Total victimisation Verbal victimisation Relational victimisation No significant change in: Aggression Peer acceptance Conflict resolution *outcomes assessed for bullying victims only (N = 78)	Skills taught through role–play, positive reinforcement and homework assignments		Effects similar for intervention and comparison groups, therefore, cannot be attributed to the intervention
Rational Emotive Behavioural Education (REBE)–ViSC/ViSC–REBE Anti–bullying Romania (Trip et al., 2015)	Grade 6 students (mean age 11.8 years) in 11 schools	Class–based CBT and anti–bullying programme Underpinning Theoretical Models: Rational emotive behavioural theory (REBE) (Ellis, 1994), social learning theory (ViSC) (Strohmeier et al., 2012) Modules: 9 REBE units (anger triggers, anger and other emotions, anger scale, consequences of anger) and 10 ViSC units (units one to eight on preventing aggressive behaviour in the classroom and units 9 and 10 on creating a common goal), experimentally delivered in alternate orders Implemented once per week through 19 units over one academic year Facilitators: external trainers	Quasi–experimental design, 2011–2012 N = 970 Intervention group: Five schools received REBE–ViSC Three schools received ViSC–REBE Controls: Received no intervention	Positive effects for: Cognitions (both intervention groups) Reducing overt anger (REBE–ViSC group only) No effects for: Internalising anger Behaviours (bullying perpetration/victimisation) *Measured at midpoint and post–intervention	Training: REBE facilitators took counselling and psychotherapy classes and attended a 4–hour presentation on REBE. ViSC trainers attended an 8–hour training session. Implementation was monitored through the self–completion of trainer evaluation forms. High fidelity reported	Programme delivered in Romanian	No significant effects for bullying perpetration/victimisation
“Skills for Life” (SFL) intervention *Selective, skill–based Chile (Guzmán et al., 2015)	“At–risk”, Second Grade elementary school students (5–8 years old) in 1,636 schools	Life skills intervention for “at–risk” students Implemented over 10 sessions (90–120 min), additional three workshops for parents and two for teachers Facilitators: psychologists (groups of six to 10 students)	Cohort longitudinal, 2010 N = 7,051	Positive effects for: Behavioural outcomes (teacher and parent reported) Academic outcomes (school attendance and promotion from third to fourth grade) *outcomes for N = 3,935 students only	Student attendance recorded: 77% attended seven to ten sessions, 23% attended zero to six sessions		Small effect sizes and large numbers lost to follow–up Linear relationship between the number of workshops attended and behavioural outcomes
Coping Skills Program *Indicated, skill–based India (Singhal et al., 2018)	Grade 8–11 students (13–18 years) in two schools	Coping Skills Program for students with subclinical depression Underpinning Theoretical Model: CBT Modules: depressive symptoms, academic stress, social problem solving, coping skills, negative cognitions Implemented once per week (45 min) over 8 weeks Facilitator: author (same gender groups of four to eight students)	Two group comparison, 2012–2013 N = 120 Controls: Received one 40–45 min psycho–educatory session	Significant improvement in: Social problem solving Coping skills Significant decrease in: Depressive symptom severity and frequency Negative thinking Academic stress *75–80% of adolescents in intervention group achieved recovery (all measures) *90–97% of controls unchanged *Effects sustained and larger at 3–month follow–up			Positive outcomes for males and females with 75–80% of adolescents achieving recovery
Yoga Columbia (Velasquez et al., 2015)	Fifth, eighth and ninth grade students in one public school	Yoga programme for the prevention of depression, anxiety and aggression Implemented once per week (2 hours) over 12 weeks, after school Facilitators: yoga teachers	RCT, mixed methods study, 2012 N = 125 Waitlist controls	Significant reduction in: Anxiety No effects for: Depression Aggression (peer assessment) Socio–emotional competencies	Activities included postures (asana), breathing exercises (pranayama), relaxation (yoga nidra), meditation. Snack provided for participants after workshops Implementation monitored through recording mean attendance (17/24 sessions) and student satisfaction. High student satisfaction reported		Positive effects for decreasing anxiety; however, small sample size

All of the studies reviewed evaluated interventions that were delivered face-to-face in the school setting, apart from the study by Anttila et al. (2019) which evaluated “DepisNet-Thai”, a web-based programme. Interventions were implemented in primary schools (N = 8), middle schools (N = 6) and secondary schools (N = 11). Participants ranged in age from 5 to 19 years and most were of lower socio-economic backgrounds living in areas of high poverty. Ten interventions were predominantly facilitated by teachers with the remaining programmes implemented by psychologists, researchers or trained external providers/community members. Training for facilitators varied in duration from 1 day to 1-week pre-intervention, with some studies reporting the provision of top-up training weekly, monthly, or midway through delivery. Two studies employed a “train-the-trainer” model, whereby teachers who received training then facilitated training for their peers (Kutcher et al., 2017; McMullen and McMullen, 2018). Fifteen interventions were developed in the implementing country. Seven were adapted versions of evidence-based programmes from HICs (Trip et al., 2015; Dang et al., 2017; Kutcher et al., 2017; Arënliu et al., 2020; Nguyen et al., 2020; McCoy et al., 2021; Seale et al., 2021), two were adaptations of programmes from other LMICs (Jegannathan et al., 2014; Karmaliani et al., 2020) and one was adapted from an evidence-based programme previously implemented in humanitarian settings (Berger et al., 2018).

Positive outcomes were found for 10 of the 13 universal mental health promotion programmes reviewed and for all five of the interventions that incorporated both promotion and prevention elements (see Tables 1 and 2), with most studies receiving a moderate or strong quality rating. Studies evaluating skill-based interventions reported improvements in participants’ life skills, including self-awareness, self-management, resilience and relationship skills, positive behaviours and decreases in peer victimisation/perpetration, while mental health literacy programmes reported increased mental health knowledge and decreased stigma among students and staff. Improvements across a range of behavioural and mental health indicators were reported for participants of SEHER (SM), a whole-school, multi-component health promotion intervention implemented by lay counsellors and the study quality was rated strong (Shinde et al., 2018), while null effects were reported for the only web-based programme reviewed (Anttila et al., 2019).

Improvements in positive outcomes, such as resilience, were found to be greater for ethnic subgroups in Sarkar et al. (2017), highlighting the potential for life skills interventions to reduce disparities among ethnic minority groups. Programmes involving families and the wider community reported improvements in attitudes to suicide (Arenas-Monreal et al., 2022) and gender attitudes and reduced physical punishment at home which was more significant for females (Karmaliani et al., 2020).

Outcomes for primary prevention programmes were more mixed. Considering anti-bullying interventions, only one study reported positive effects for one victimisation indicator for participants of the 4-week ViSC programme (Arënliu et al., 2020). Two of the three targeted interventions reviewed reported positive outcomes for “at-risk” participants of a Skills for Life programme (Guzmán et al., 2015) and students with subclinical depression who participated in a coping skills programme (Singhal et al., 2018).

Mental health outcomes were measured by 14 studies and considered happiness, life satisfaction, internalising mental health problems such as depression and anxiety and behavioural externalising problems. Most of the studies reporting on positive mental health outcomes evaluated skill-based interventions. Few studies measured academic outcomes with only two reporting positive programme effects. For participants of the teacher-led, ESPS stress-reduction programme, improvements in academic performance were found to be more significant for children living in an orphanage, highlighting the potential for programmes to reduce educational disparities among relatively disadvantaged young people (Berger et al., 2018). Differential programme effects according to gender, level of adversity and risk were reported by some studies (see Tables 1 and 2 for details).

All 19 effective studies reported on some aspects of programme implementation, while 15 referenced monitoring fidelity of implementation. Quality of delivery was the most widely measured and reported domain and was monitored through session observations, audio recordings of sessions and facilitator reports. Seven effective studies reported on adherence, three on dosage and six on participant responsiveness. In terms of programme differentiation, shortening programme durations (Arënliu et al., 2020) and the delivery of whole-school interventions as class-based curricula (Dang et al., 2017; Arënliu et al., 2020), may have adversely affected outcomes.

Most studies provided details on modules delivered. Eleven studies outlined the intervention’s underpinning theoretical model. Sessions were generally delivered weekly in participatory group-format, and most were between 45 and 60 minutes long (N = 10). Modules of skill-based interventions focussed on communication, self-awareness, self-control, relationships and coping skills. One study also included general health modules (Sarkar et al., 2017), while three studies facilitated modules for families and/or school staff. Mental health literacy interventions included modules on positive mental health, stigma, understanding mental health and information on supports, while anti-bullying interventions focussed on developing social skills and recognising bullying. SEHER also incorporated general health modules in addition to modules on mental health, bullying, substance use, gender equality and violence (Shinde et al., 2018).

Several moderators of effective implementation were identified in the reviewed studies. The main implementation barriers that were reported included large class sizes (Leventhal et al., 2015; McMullen and Eaton, 2021), time constraints (Shinde et al., 2018; Shinde et al., 2020), lack of ongoing support for teachers (Nguyen et al., 2020) and limited physical space (Arënliu et al., 2020). High teacher turnover due to community unrest was reported to have adversely affected implementation by McCoy et al. (2021), highlighting the importance of considering the effects of broader contextual and societal factors on programme implementation. Facilitators of implementation included adequate time for session delivery through the provision of dedicated timetable slots, after-school delivery or implementation in non-exam classes, the support of management and staff, adequate resources including physical space and a complementary school ethos. At a macro-level, effective partnerships, for example, with Non-Governmental Organisations (NGOs) and communities, were highlighted as being key to programme delivery and sustainability.

Eleven studies referenced the cultural adaptation of programmes, with only four studies reporting details (Berger et al., 2018; Nguyen et al., 2020; McCoy et al., 2021; Seale et al., 2021). The adaptation process involved an initial assessment of the original programme with all stakeholders, subsequent adaptations including to language and content, piloting to check for cultural appropriacy and final adjustments following consultation with the implementation team. Youth involvement in the process was reported as facilitating natural cultural adaptation by Leventhal et al. (2015), while involving the programme developers helped to ensure that the core components of the programme were retained (Dang et al., 2017). Eight studies reported the translation of programme language, while Seale et al. (2021) highlighted that the delivery of GROW through English excluded some younger students and considered its future delivery in local Zambian dialects. Some studies referenced adding culturally relevant content to programmes to increase participant responsiveness. Traditional folk stories and culturally applicable concepts like “shikamoo”, which refers to respect for the elderly, were incorporated into the ESPS programme (Berger et al., 2018), while Seale et al. (2021) reported incorporating local customs and a programme motto, “never give up, never surrender”, to GROW. Other studies rephrased terms such as “mental illness” to “mental health problems” and added references to national celebrities (Nguyen et al., 2020), sports (McCoy et al., 2021) and dietary practices (Sarkar et al., 2017). The involvement of key community members, such as pastors in programme delivery (Seale et al., 2021), and local healers who blessed the ESPS programme before it commenced (Berger et al., 2018), was considered integral to the local acceptance of programmes.

## Discussion

This scoping review sought to map the peer-reviewed and grey literature published from 2014–2022 on the process of implementing effective mental health promotion and prevention interventions in schools in LMICs. A total of 27 studies evaluating 25 school-based mental health interventions were identified. Although the studies spanned a wide geographical area consistent with other reviews (Barry et al., 2013; Chuecas et al., 2022), a small number (N = 4) were from low-income countries. The increasing number of programmes with a mental health promotion focus and the number of interventions implemented in primary schools (N = 8) is encouraging and highlights the feasibility of implementing school-based programmes in LMIC settings. Most study designs were RCT, cRCT or quasi-experimental; however, mirroring findings from previous reviews (Durlak et al., 2011; Bradshaw et al., 2021), study quality varied and only a few studies had longer follow-up periods, which would allow for better assessment of whether mental health outcomes were sustained. Reporting on implementation varied greatly between studies, and no study comprehensively measured all five domains of implementation. Overall, 15 effective studies reported implementation fidelity, with quality of delivery being the most widely measured domain. Consistent with previous literature (Durlak et al., 2011; Dowling and Barry, 2020), more significant positive effects were found when implementation quality was high. Eleven studies referenced the cultural adaptation of programmes, although as discussed in existing literature (Castro-Olivo, 2017; Bradshaw et al., 2021), few (N = 4) provided sufficient detail for replication or referenced employing an evidence-based adaptation framework to guide the process.

Of the interventions reviewed, universal skill-based mental health promotion programmes and skill-based targeted interventions for “at-risk” students and those with subclinical depression positively impacted young people’s life-skills and to a lesser extent mental health outcomes. As the literature suggests, there is a need to measure and report on mental health outcomes and to implement interventions of longer duration for significant mental health impacts to be achieved (Das et al., 2016; Singla et al., 2020). Universal interventions that adopted a whole-school approach or involved families and communities produced more sustained effects, created environments supportive of positive mental health and impacted the wider determinants of young people’s mental health (Shinde et al., 2018; Karmaliani et al., 2020). Programmes that delivered general health modules in addition to life-skills and mental health modules yielded positive outcomes across a range of well-being and mental health indicators. In considering the largely null effects of anti-bullying interventions reviewed, preference should be given to longer-term, teacher-led whole-school approaches due to the complex nature of bullying (Arënliu et al., 2020), and its association with poorer academic outcomes and mental health difficulties (Sivaraman et al., 2019). Previous literature has pointed to the potential for school-based mental health literacy programmes to decrease stigma in communities (Jorm, 2012) and both of the teacher-led mental health literacy interventions reviewed reported positive outcomes for increasing student and staff knowledge on mental health and decreasing stigma. Of note also were the differential programme effects according to gender, adversity and risk, endorsing the need to consider gender-specific components and comprehensive mental health initiatives in schools that incorporate both universal and targeted programmes (Barry et al., 2013; Singla et al., 2020).

This research aimed to investigate the implementation process of reviewed programmes, and consistent with previous reviews, higher implementation fidelity yielded more positive programme effects. This is best demonstrated by comparing the positive, sustained effects of the multi-component SEHER when implemented by lay counsellors (SM) with the null effects when implemented by class teachers (TSM), who reported inadequate support and time for delivery (Shinde et al., 2018, 2020). Previous reviews have pointed to the benefits of teacher-led, mental health interventions in low-resource school settings in terms of improved relationships with students, mental health and academic outcomes and cost-effectiveness (Barry et al., 2013; Fenwick-Smith et al., 2018; Gimba et al., 2020). However, as reported in Shinde et al. (2018), training and support for teachers and addressing any barriers to implementation is crucial to positive outcomes. Studies reporting a lack of physical space and materials for programme delivery highlighted the importance of careful planning and implementation support at the local level. Employing an implementation framework, such as the Consolidated Framework for Implementation Research (Damschroder et al., 2022), can help to guide the implementation process and map systems-wide barriers and facilitators to implementation. Finally, effective partnerships are considered crucial to health promotion (Corbin et al., 2016), and strong partnerships between schools, NGOs and partner universities were highlighted by many studies as facilitating the development, implementation and sustainability of interventions.

The potential for evidence-based interventions from HICs and other settings to be culturally adapted and effectively delivered and scaled-up in LMICs has been highlighted previously (Bradshaw et al., 2021; Jannesari et al., 2021), and several studies in this review referenced the role of cultural adaptation in positive outcomes and the external validity of programmes. However, few of the reviewed studies reported in any detail on the cultural adaptation process. Of the studies that did provide details, the involvement of key community figures in the adaptation process was considered to promote programme acceptance, while the input of programme developers was viewed as essential to ensuring that the core components of the programme were retained. While involving students in programme development was considered important in facilitating the natural cultural adaptation of interventions by Leventhal et al. (2015), few other studies reported the involvement of young people in the process. Of the studies that reported positive outcomes and provided detail on the cultural adaptation of interventions, piloting the programme with young people to check for cultural appropriacy in advance of implementation emerged as a key stage of the process. Utilising a cultural adaptation framework such as the four-step heuristic framework for cultural adaptation (Barrera and Castro, 2006) or the eight-domain EVM (Bernal et al., 1995) could help guide the process and reporting on it in detail would allow for replication.

The findings from this scoping review show that school-based mental health promotion and prevention programmes, in particular interventions that focus on the promotion of positive mental health, are effective in increasing life-skills and prosocial behaviours and decreasing mental health symptoms and stigma for young people when implemented to a high level. The dearth of studies reporting on academic outcomes makes accurate conclusions on the impact of interventions on students’ learning in LMIC schools difficult to reach. However, previous reviews from HICs have pointed to the positive impact of teacher-led programmes on academic achievement and consequently employment opportunities in adulthood (Durlak et al., 2011; Taylor et al., 2017).

This review highlights the critical importance of high-quality implementation of mental health promotion and prevention programmes in LMIC schools. The findings reinforce the need for more detailed research in this area, including measuring and reporting on implementation and investigating and addressing barriers to effective implementation so that programmes can be sustained outside of research conditions and scaled-up at a country level. The findings on cultural adaptation contribute to the dearth of literature in this area and endorse its crucial role in the local acceptance of programmes. The review also highlights the need for studies to provide adequate detail on the adaptation process. Metrics to determine the strength of evidence in relation to implementation effectiveness and cultural adaptation would provide useful information in addition to existing metrics assessing the quality of study design.

## Strengths and limitations

This review maps the evidence of mental health promotion and prevention interventions in both primary and secondary schools in LMICs from 2014 to 2022. A comprehensive search strategy was employed to search numerous electronic databases and grey literature sources. The strengths of the review lie in the inclusion of all study designs, which detailed a variety of interventions and outcomes, giving the review depth.

Considering the study limitations, a more extensive grey literature search could have yielded many more relevant studies. In addition, due to time constraints, interventions implemented in humanitarian contexts were deemed special cases and excluded. Only studies published in English were included, thereby excluding many potentially relevant studies published in other languages.

## Conclusion

This scoping review mapped the evidence of mental health promotion and prevention interventions in both primary and secondary schools in LMICs, examining the processes of implementation and cultural adaptation. Findings were generally positive and strengthen the evidence base of the effectiveness of school-based interventions in promoting young people’s mental health and well-being. Many studies were considered moderate to strong quality and several employed RCT or cRCT designs. The growing number of studies reporting on implementation is encouraging; however, there is a need for more stringent monitoring and reporting on implementation fidelity and the core components of programmes. Likewise, more robust research is needed on the cultural adaptation of interventions. In relation to intervention outcomes, measures of social and emotional well-being and positive mental health, academic outcomes, cost-effectiveness and longer-term follow-up periods are required to further strengthen the evidence base.

Current global policy frameworks endorse the need for a population approach to mental health promotion in key settings across the life-course, including in schools (UNICEF, 2021; WHO, 2021a). The number of effective interventions reviewed in this paper is promising and highlights the feasibility of implementing school-based programmes in LMIC settings. However, ensuring that programmes are culturally appropriate and implemented effectively will be key to the sustainability and scaling-up of interventions at a national level in order to improve the mental health and well-being of young people living in LMICs.

## Supporting information

Harte and Barry supplementary material Harte and Barry supplementary material

## Data Availability

The authors confirm that the data supporting the findings of this study are available within the article and its supplementary material.

## Supplementary material

The supplementary material for this article can be found at http://doi.org/10.1017/gmh.2024.48.
