# Supplementary material for: A scoping review of the implementation and cultural adaptation of school-based mental health promotion and prevention interventions in low-and middle-income countries
Source: Glob Ment Health (Camb). 2024 Apr 12;11:e55. doi: 10.1017/gmh.2024.48 (PMC11094552; doi:10.1017/gmh.2024.48)
Supplement: Harte and Barry supplementary material [file S2054425124000487sup001.pdf]

Table S1: Search Terms used across all databases

Population Terms	Setting Terms	Positive Mental Health Terms	Negative Mental Health Terms	Programme Terms	Context Terms
"School pupil" OR	School* OR	"Mental health" OR	Suicide OR	Intervention* OR	"Developing countr*" OR
"School child"	Classroom*	Wellbeing	Depression	Education	"Developing world"
Schoolchild*	education	"Social wellbeing"	Anxiety	Support	"Developing nation"
"Young people"	"Alternative education"	"Emotional wellbeing"	Behaviour*	Training	"Less developed countr*"
Child*		Coping	Conduct	Therapy	"Underdeveloped countr*"
Youth*		"Emotional health"	"Self-harm"	Promoti*	"Global south"
Adolescent*		"Mental capital"	Prevent*	Prevent*	"Third world countr*"
Student*		Psychosocial	Bullying	Universal	"Middle-income countr*"
Teenager*		Psychoeducation		Indicated	"Low-income countr*"
Juvenile*		"Positive psychology"		Selective	"Low- and middle- income countr*"
		"Well-being"		Program*	LMIC
		"Social emotional"		"At risk"	
		"Mental health literacy"		Subclinical	
		Mindfulness			
		"Youth development"			

NOTE: **Denotes any ending including singular/plural, "" denotes phrase search*

Table S2: Search Strategy used for CINAHL Database on 17/05/2022

**Search 1**
TI ("school pupil" OR "school child*" OR schoolchild* OR "young people" OR child* OR adolescent* OR youth OR student* OR teenager* OR juvenile) OR AB ("school pupil" OR "school child*" OR schoolchild* OR "young people" OR child* OR adolescent* OR youth OR student* OR teenager* OR juvenile OR adolescence)
AND
TI (school* OR classroom OR "alternative education" OR educat*) OR AB (school* OR classroom OR "alternative education" OR educat*)
AND
TI ("mental health" OR "well being" OR "well-being" OR wellbeing OR social OR emotional OR coping OR communication OR resilience OR "emotional health" OR "mental capital" OR psychosocial OR psychoeducation OR "positive psychology" OR "social emotional" OR "mental health literacy" OR mindfulness OR "youth development") OR AB ("mental health" OR "well being" OR "well-being" OR wellbeing OR social OR emotional OR coping OR communication OR resilience OR "emotional health" OR "mental capital" OR psychosocial OR psychoeducation OR "positive psychology" OR "social emotional" OR "mental health literacy" OR mindfulness OR "youth development")
AND
TI (program* OR "school based intervention" OR intervention OR educat* OR support OR training OR treatment OR therapy OR promoti* OR preventi* OR universal OR indicated OR selective OR "at risk" OR "sub-clinical") OR AB (program* OR "school based intervention" OR intervention OR educat* OR support OR training OR treatment OR therapy OR promoti* OR preventi* OR universal OR indicated OR selective OR "at risk" OR "sub-clinical")
AND
TI ("developing countr*" OR "developing world" OR "developing nation" OR "less developed countr*" OR "underdeveloped countr*" OR "global south" OR "third world countr*" OR "middle income countr*" OR "low income countr*" OR "LMIC" OR "low and middle income countr*" OR "developing countries") OR AB ("developing countr*" OR "developing world" OR "developing nation" OR "less developed countr*" OR "underdeveloped countr*" OR "global south" OR "third world countr*" OR "middle income countr*" OR "low income countr*" OR "LMIC" OR "low and middle income countr*" OR "developing countries")
**Search 2**
TI ("school pupil" OR "school child*" OR schoolchild* OR "young people" OR child* OR adolescent* OR youth OR student* OR teenager* OR juvenile) OR AB ("school pupil" OR

"school child*" OR schoolchild* OR "young people" OR child* OR adolescent* OR youth OR student* OR teenager* OR juvenile OR adolescence)
AND
TI (school* OR classroom OR "alternative education" OR educat*) OR AB (school* OR classroom OR "alternative education" OR educat*)
AND
TI (bullying OR "suicide prevention" OR suicide OR depression OR anxiety OR behavioural OR behavioral OR conduct OR "self harm") OR AB (bullying OR "suicide prevention" OR suicide OR depression OR anxiety OR behavioural OR behavioral OR conduct OR "self harm")
AND
TI (program* OR "school based intervention" OR intervention OR educat* OR support OR training OR treatment OR therapy OR promoti* OR preventi* OR universal OR indicated OR selective OR "at risk" OR "sub-clinical”) OR AB (program* OR "school based intervention" OR intervention OR educat* OR support OR training OR treatment OR therapy OR promoti* OR preventi* OR universal OR indicated OR selective OR "at risk" OR "sub-clinical")
AND
TI ("developing countr*" OR "developing world" OR "developing nation" OR "less developed countr*" OR "underdeveloped countr*" OR "global south" OR "third world countr*" OR "middle income countr*" OR "low income countr*" OR "LMIC" OR "low and middle income countr*" OR "developing countries") OR AB ("developing countr*" OR "developing world" OR "developing nation" OR "less developed countr*" OR "underdeveloped countr*" OR "global south" OR "third world countr*" OR "middle income countr*" OR "low income countr*" OR "LMIC" OR "low and middle income countr*" OR "developing countries")
