# Supplementary material for: A scoping review of the implementation and cultural adaptation of school-based mental health promotion and prevention interventions in low-and middle-income countries
Source: Glob Ment Health (Camb). 2024 Apr 12;11:e55. doi: 10.1017/gmh.2024.48 (PMC11094552; doi:10.1017/gmh.2024.48)
Supplement: Peer review history

# Editor report: Recommendation: A scoping review of the implementation and cultural adaptation of school-based mental health promotion and prevention interventions in low-and middle-income countries — R0/PR2

**DOI:** 10.1017/gmh.2024.48.pr2

**Affiliations:** Psychiatry, Makerere University CHS, Uganda

No accompanying comment.

# Editor report: Decision: A scoping review of the implementation and cultural adaptation of school-based mental health promotion and prevention interventions in low-and middle-income countries — R0/PR3

**DOI:** 10.1017/gmh.2024.48.pr3

**Affiliations:** London School of Hygiene & Tropical Medicine, United Kingdom of Great Britain and Northern Ireland

No accompanying comment.

# Editor report: Recommendation: A scoping review of the implementation and cultural adaptation of school-based mental health promotion and prevention interventions in low-and middle-income countries — R1/PR5

**DOI:** 10.1017/gmh.2024.48.pr5

**Affiliations:** Psychiatry, Makerere University CHS, Uganda

The authors have addressed all the reviewers' comments

# Editor report: Decision: A scoping review of the implementation and cultural adaptation of school-based mental health promotion and prevention interventions in low-and middle-income countries — R1/PR6

**DOI:** 10.1017/gmh.2024.48.pr6

**Affiliations:** London School of Hygiene & Tropical Medicine, United Kingdom of Great Britain and Northern Ireland

No accompanying comment.
